# Supplementary material for: The interactome of intact mitochondria by cross-linking mass spectrometry provides evidence for coexisting respiratory supercomplexes
Source: Mol Cell Proteomics. 2017 Dec 8;17(2):216–32. doi: 10.1074/mcp.RA117.000470 (PMC5795388; doi:10.1074/mcp.RA117.000470)
Supplement: Supplemental Data [file supp_RA117.000470_133922_0_supp_23978_fzffwf.zip › spectra_annotation/mito_DR_spectra_annotation/346-1-10-1-11-1.pdf]

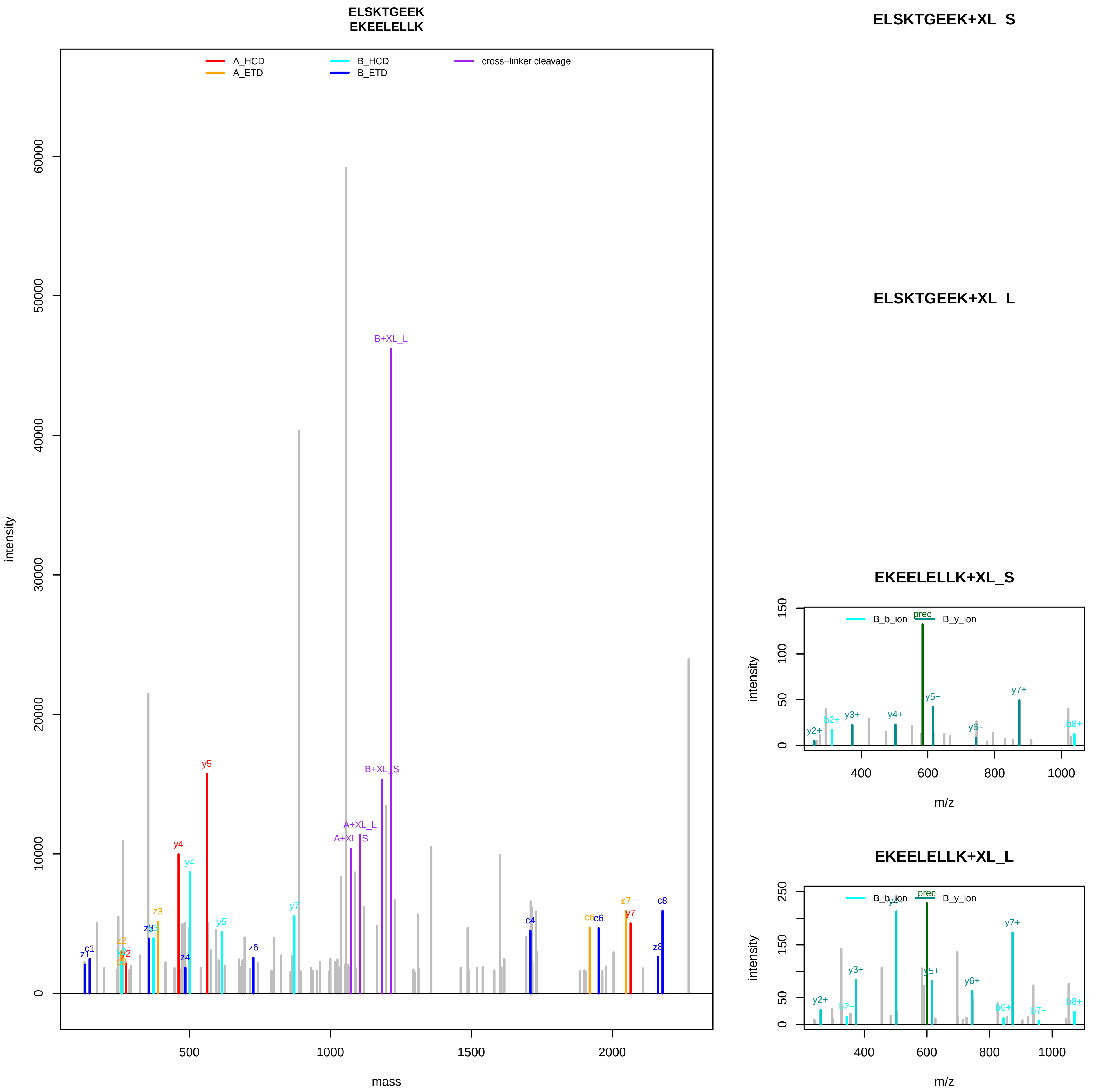
ELSKTGEEK
EKEELELLK
A_HCD
A_ETD
B_HCD
B_ETD
cross−linker cleavage
intensity
mass
0
10000
20000
30000
40000
50000
60000
500
1000
1500
2000
B+XL_L
B+XL_S
A+XL_L
A+XL_S
y2
y4
y5
y7
y4
y5
y7
z1
c1
z2
c2
z3
z3
z4
z6
c4
c6
c6
z7
z8
c8
ELSKTGEEK+XL_S
ELSKTGEEK+XL_L
EKEELELLK+XL_S
B_b_ion
B_y_ion
prec
y2+
b2+
y3+
y4+
y5+
y6+
y7+
b8+
m/z
400
600
800
1000
0
50
100
150
EKEELELLK+XL_L
B_b_ion
B_y_ion
prec
y2+
b2+
y3+
y4+
y5+
y6+
b6+
y7+
b7+
b8+
m/z
250
150
50
0
